# *C. albicans* growth, transition, biofilm formation, and gene expression modulation by antimicrobial decapeptide KSL-W

**DOI:** 10.1186/1471-2180-13-246

**Published:** 2013-11-07

**Authors:** Simon Theberge, Abdelhabib Semlali, Abdullah Alamri, Kai P Leung, Mahmoud Rouabhia

**Affiliations:** 1 Oral Ecology Research Group, Faculty of Dentistry, Laval University, 2420, rue de la Terrasse, Quebec G1V 0A6, QC, Canada; 2 Department of Biochemistry, Genome Research Chair, College of Science King Saud University, Riyadh, Kingdom of Saudi Arabia; 3 Dental and Trauma Research Detachment, US Army Institute of Surgical Research, JBSA Fort Sam Houston, San Antonio, TX, USA

**Keywords:** Antimicrobial peptide, KSL-W, *C. albicans*, Growth, Hyphae, Gene, EFG1, NRG1, HWP1, SAPs

## Abstract

**Background:**

Antimicrobial peptides have been the focus of much research over the last decade because of their effectiveness and broad-spectrum activity against microbial pathogens. These peptides also participate in inflammation and the innate host defense system by modulating the immune function that promotes immune cell adhesion and migration as well as the respiratory burst, which makes them even more attractive as therapeutic agents. This has led to the synthesis of various antimicrobial peptides, including KSL-W (KKVVFWVKFK-NH_2_), for potential clinical use. Because this peptide displays antimicrobial activity against bacteria, we sought to determine its antifungal effect on *C. albicans*. Growth, hyphal form, biofilm formation, and degradation were thus examined along with EFG1, NRG1, EAP1, HWP1, and SAP 2-4-5-6 gene expression by quantitative RT-PCR.

**Results:**

This study demonstrates that KSL-W markedly reduced *C. albicans* growth at both early and late incubation times. The significant effect of KSL-W on *C. albicans* growth was observed beginning at 10 μg/ml after 5 h of contact by reducing *C. albicans* transition and at 25 μg/ml by completely inhibiting *C. albicans* transition. Cultured *C. albicans* under biofilm-inducing conditions revealed that both KSL-W and amphotericin B significantly decreased biofilm formation at 2, 4, and 6 days of culture. KSL-W also disrupted mature *C. albicans* biofilms. The effect of KSL-W on *C. albicans* growth, transition, and biofilm formation/disruption may thus occur through gene modulation, as the expression of various genes involved in *C. albicans* growth, transition and biofilm formation were all downregulated when *C. albicans* was treated with KSL-W. The effect was greater when *C. albicans* was cultured under hyphae-inducing conditions.

**Conclusions:**

These data provide new insight into the efficacy of KSL-W against *C. albicans* and its potential use as an antifungal therapy.

## Background

The innate defense system plays a key role in protecting the host against microorganism-fueled infections such as candidiasis caused by *Candida albicans. C. albicans* colonizes several body sites, including the oral cavity; however, as a commensal organism, it causes no apparent damage or inflammation in the surrounding tissue [1,2]. *C. albicans* is a polymorphic organism that adheres to different surfaces in the body and can grow as yeast, pseudohyphae, and hyphae [3], usually in the form of biofilm. *C. albicans* transition, biofilm formation, and pathogenesis are under the control of various genes. The *HWP1* gene encodes the hyphal cell wall protein, which is a hyphal-specific adhesin that is essential to biofilm formation [4]. The involvement of *HWP1* in *C. albicans* adhesion is supported by the *EAP1* gene which encodes a glucan-crosslinked cell wall protein (adhesin Eap1p). Together, these components mediate *C. albicans* adhesion to various surfaces, such as epithelial cells and polystyrene [5]. Like many other genes, *HWP1* and *EAP1* are downstream effectors of EFG1 and NRG1 as transcription factors [6,7]. *EFG1* mutant strain has been shown to exhibit defects in growth, biofilm formation, and virulence [8], while NRG1 represses filamentous growth [3]. This occurs through the DNA binding protein Nrg1p in conjunction with the global transcriptional repressor Tup1p to suppress hyphal formation. Elevated NRG1 expression represses the expression of a number of hypha-specific genes, although NRG1 downregulation is associated with *C. albicans* filaments [3].

*C. albicans* virulence is also mediated by proteolytic enzymes, including secreted aspartyl proteinases (SAPs) [9,10]. The contribution of SAPs in *C. albicans* adherence, tissue damage, and evasion of host immune responses has been reported [9]. SAP2 is crucial to *C. albicans* growth in protein-containing media [11]. SAP1 and SAP3 are expressed during phenotypic switching [12,13], while SAP4, SAP5, and SAP6 are expressed upon hyphal formation [14], and SAPs 1-6 and 9-10 are involved in the adhesion mechanism to host cells [15].

To control *C. albicans* pathogenesis, the host innate immunity uses small molecules such as proteins and peptides that display a broad antimicrobial spectrum. The number of identified potentially antimicrobial peptides is significant and continues to increase [16]. Antimicrobial peptides often possess common attributes, such as small size, an overall positive charge, and amphipathicity [17,18]; however, they also fall into a number of distinctively diverse groups, including α-helical peptides, β-sheet peptides, peptides with mixed α-helical and β-sheet structures, extended peptides, and peptides enriched in specific amino acids [16].

In humans, epithelial cells and neutrophils are the most important cells producing antimicrobial peptides [19,20]. These peptides are most often antibacterial, although antifungal activity has also been reported [16,21]. The major peptide groups known to date are the histatins, cathelicidins, defensins, and lactoferricins [22]. The antimicrobial activity of these peptides has been reported by different *in vitro* and *in vivo* studies [19,20,22]. Their complex role as well as their contribution to host defenses may be related to the functional interrelationship between innate and adaptive immunity [23,24].

The interest in antimicrobial peptides lies in the possible resistance of microorganisms to conventional antimicrobial strategies used against microbial pathogens in both agriculture and medicine [25,26]. Natural antimicrobial peptides are necessary in the control of microbial infections. For example, the use of AMPs provided protection against such microbial pathogens as fungal pathogens, with no reported effect on the host [27,28]. Based on these promising data, a number of synthetic AMPs have been designed to overcome microbial infections [29]. In the pursuit of a novel alternative antifungal treatment, we developed a synthetic α-helical antimicrobial decapeptide, KSL (KKVVFKVKFK), and its analogue KSL-W (KKVVFWVKFK) [30].

The efficacy of KSL on a wide range of microorganisms has been established [31-33], as well as its ability to disrupt oral biofilm growth [34]. KSL-W, a recently synthesized KSL analogue, was shown to display improved stability in simulated oral and gastric conditions with *in vitro* preserved antimicrobial activity [30]. Furthermore, combined with sub-inhibitory concentrations of benzalkonium chloride, a known cationic surface-active agent [35], KSL was shown to significantly promote bacterial biofilm susceptibility. We also recently demonstrated that KSL-W had a selective effect on *C. albicans* growth, while exhibiting no toxic effect on epithelial cells [36].

As this KSL-W analogue displays a wide range of microbicidal activities, effectively kills bacteria, controls biofilm formation, and destroys intact biofilms, we hypothesized that KSL-W may also possess antifungal potential. Our goal was thus to investigate the ability of KSL-W to inhibit *C. albicans* growth and transition from blastospore to hyphal form. The action of KSL-W on biofilm formation/disruption was also assessed. Finally, we examined the effect of KSL-W on various *C. albicans* genes involved in its growth, transition, and virulence.

## Results

### Antimicrobial peptide KSL-W reduced *C. albicans* growth and transition from blastospore to hyphal form

**Figure 1:**
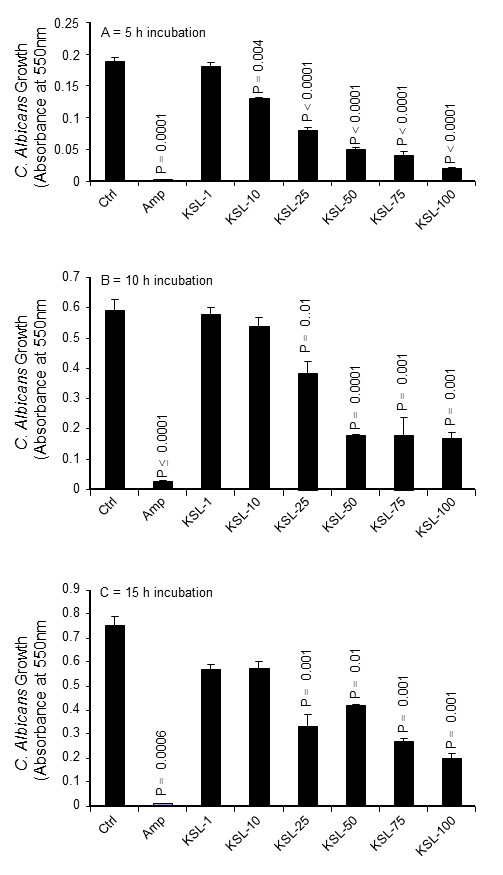
**KSL-W inhibited *C. albicans *growth.** The yeast was cultured in Sabouraud supplemented medium with or without KSL-W at various concentrations. The cultures were maintained for 5, 10, and 15 h at 37°C, after which time an MTT assay was performed for each culture condition. The growth was plotted as means ± SD of the absorbance at 550 nm. **(A)***C. albicans* growth with KSL-W for 5 h; **(B)***C. albicans* growth with KSL-W for 10 h; and **(C)***C. albicans* growth with KSL-W for 15 h. The levels of significance for *C. albicans* growth in the presence or not of KSL-W or amphotericin B (10 μg/ml) were considered significant at *P* < 0 · 05.

*C. albicans* cultures were incubated with KSL-W for 5, 10, and 15 h to determine whether this antimicrobial peptide had any adverse effect on *C. albicans* growth. As shown in Figure 1, KSL-W significantly reduced *C. albicans* proliferation. After 5 h of contact with KSL-W, the growth inhibition of *C. albicans* was between 30 and 80%, depending on the concentration of KSL-W used (Figure 1A). After 10 h of contact with KSL-W, growth inhibition was significant, beginning at 25 μg/ml (Figure 1B). At later culture periods, *C. albicans* growth continued to be significantly affected by the presence of KSL-W (Figure 1C). Indeed, with 25 μg/ml of KSL-W, *C. albicans* growth was almost half that in the controls (non-treated *C. albicans* cultures), and with 100 μg/ml of KSL-W, *C. albicans* growth was reduced by almost 60%. It is interesting to note that KSL-W in as low as 25 μg/ml was effective at both the early and late culture periods.

**Figure 2:**
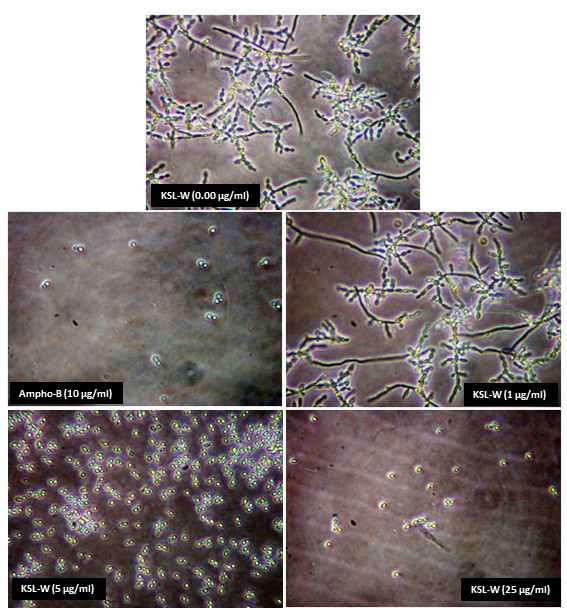
**KSL-W inhibited *C. albicans *yeast-to-hyphae transition.***C. albicans* was cultured in Sabouraud medium containing 10% fetal bovine serum with or without KSL-W at various concentrations and was maintained for 4 and 8 h at 37°C. After each time point, the cultures were observed under an inverted microscope and photographed. Representative photos of the morphological changes after 4 h of culture are presented.

As KSL-W contributed to *C. albicans* growth inhibition, we hypothesized that it would also downregulate *C. albicans* transition from yeast form to hyphal phenotype. Yeast cultures supplemented with 10% FBS and the KSL-W peptide were maintained for various incubation periods. As shown in Figure 2, germ tube formation was inhibited as early as 4 h following exposure to the peptide, compared to that in the cultures incubated in the absence of KSL-W. Of interest is the elevated number of *C. albicans* hyphal forms in the negative control culture (no KSL-W or amphotericin B) compared to the low number in the presence of KSL-W. The effect of this antimicrobial peptide on *C. albicans* transition was also dose-dependent: at 1 μg/ml, a significant number of hyphal forms remained, and at only 5 μg/ml of KSL-W, *C. albicans* transition was completely inhibited (Figure 2). Semi-quantitative analyses using inverted microscope observations to estimate the hyphal forms confirmed the inhibited *C. albicans* transition when treated with KSL-W (Table 1). The density of the hyphae was reduced as early as 4 h of contact with 5 μg/ml of KSL-W. This effect was further supported when *C. albicans* was placed in contact with KSL-W for 8 h (Table 1), thus confirming that KSL-W downregulated *C. albicans* growth and transition.

**Table 1:** **Estimation of hyphae forms in the *C. albicans *** **culture**

**Active molecules**	**Concentration (μg/mL)**	**Transition at 4 h**	**Transition at 8 h**
Negative control	0	++	++
KSL-W	1	++	++
	5	-	-
	10	-	-
	15	-	-
	25	-	-
	100	-	-
Amphotericin B	1	-	-

This Table depicts the presence of hyphae following 4 and 8 h treatments of *C. albicans* with and without KSL-W or amphotericin B. (–) refers to the absence hyphae form, and (++) refers to the presence high number of hyphae forms. These data were estimated after evaluation over 20 fields from each culture condition, by two independent and blinded examiners.

### KSL-W reduced *C. albicans* biofilm formation

**Figure 3:**
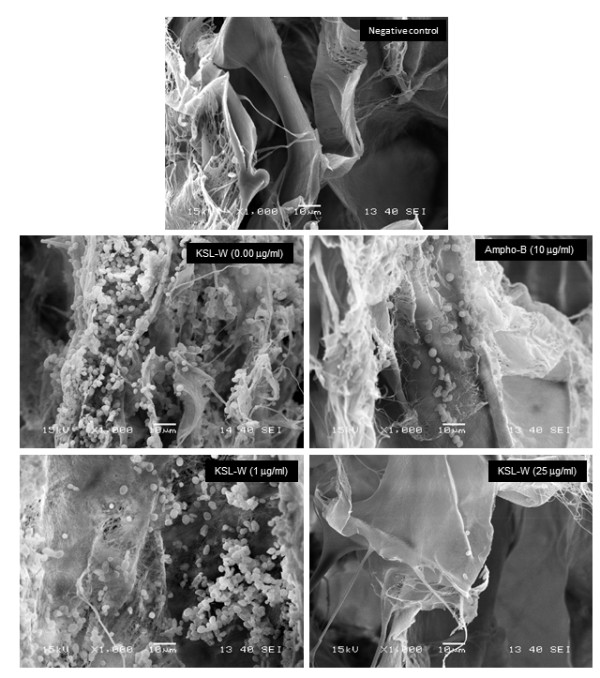
**Scanning electron microscope analyses of the biofilm formation.***C. albicans* was cultured in Sabouraud medium with or without KSL-W at various concentrations for 4 days in a porous 3D collagen scaffold. Cultures in the presence of amphotericin B (10 μg/ml) were used as the positive controls. Following incubation, the samples were prepared as described in the Methods section and were observed under a scanning electron microscope. Negative control refers to the non-seeded scaffolds.

**Figure 4:**
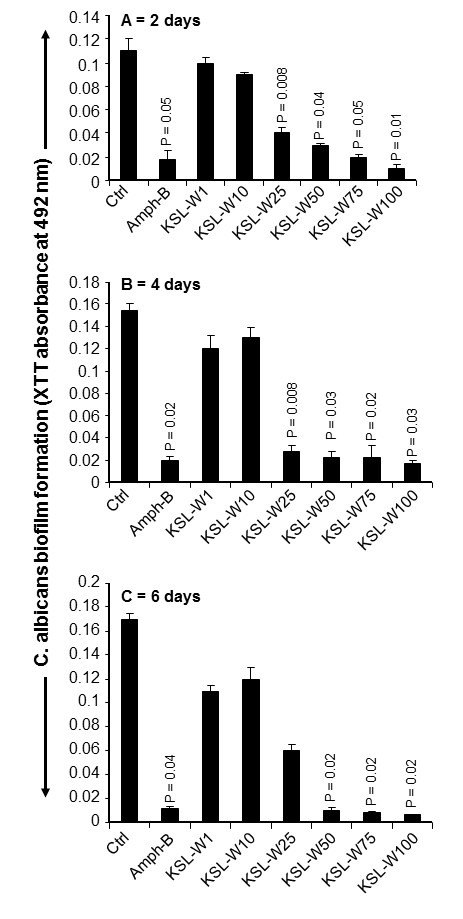
**Quantitative measurement of the reduced biofilm formation with KSL-W.***C. albicans* was cultured on a 3D porous scaffold in the presence of KSL-W for 2, 4, and 6 days. After each culture period, the samples were supplemented with XTT solution and incubated for 5 h at 37°C. The absorbance at 450 nm was measured to quantify XTT metabolic product intensity proportional to the number of viable cells. **(A)** 2 days; **(B)** 4 days; **(C)** 6 days. Results are means ± SD for three different separate experiments.

As KSL-W contributed to reducing *C. albicans* growth and transition, we sought to determine whether it also displayed inhibitory activity against *C. albicans* biofilm formation. Using a biofilm-promoting scaffold, SEM analyses, and an XTT assay, we were able to demonstrate the inhibitory effect of KSL-W on biofilm formation (Figure 3). SEM analyses revealed a significant density of *C. albicans* in the untreated culture, compared to a lower density in the scaffold in the presence of KSL-W (1 and 25 μg/ml) after 4 days of culture. The decreases obtained with the KSL-W, particularly at 25 μg/ml (Figure 3), were comparable to that obtained with amphotericin B at 10 μg/ml. To confirm these observations, we performed quantitative analyses using the XTT assay. Figure 4A shows that after 2 days of culture, KSL-W was able to inhibit biofilm formation. This inhibitory effect was observed beginning at 25 μg/ml of KSL-W. At concentrations of 50, 75, and 100 μg/ml of KSL-W, the inhibition of *C. albicans* biofilm formation was comparable to that caused by amphotericin B at 10 μg/ml. Similar results were obtained after 4 days (Figure 4B) and 6 days (Figure 4C) of culture for biofilm formation with a persistent inhibitory effect of KSL-W on *C. albicans* biofilm formation.

### KSL-W disrupted mature *C. albicans* biofilms

**Figure 5:**
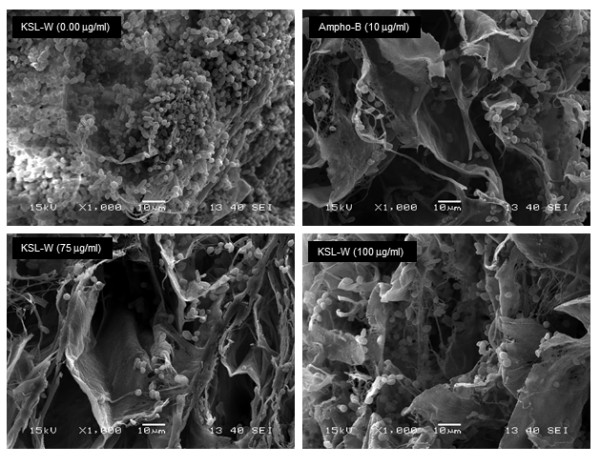
**Biofilm ultrastructure following KSL-W treatment.***C. albicans* was cultured in Sabouraud medium without KSL-W for 6 days to promote biofilm formation and maturation. The resulting biofilms were then treated or not with KSL-W or amphotericin B for 6 days, with medium and peptide refreshing every 2 days. Following incubation, the samples were prepared as described in the Methods section and observed under a scanning electron microscope.

**Figure 6:**
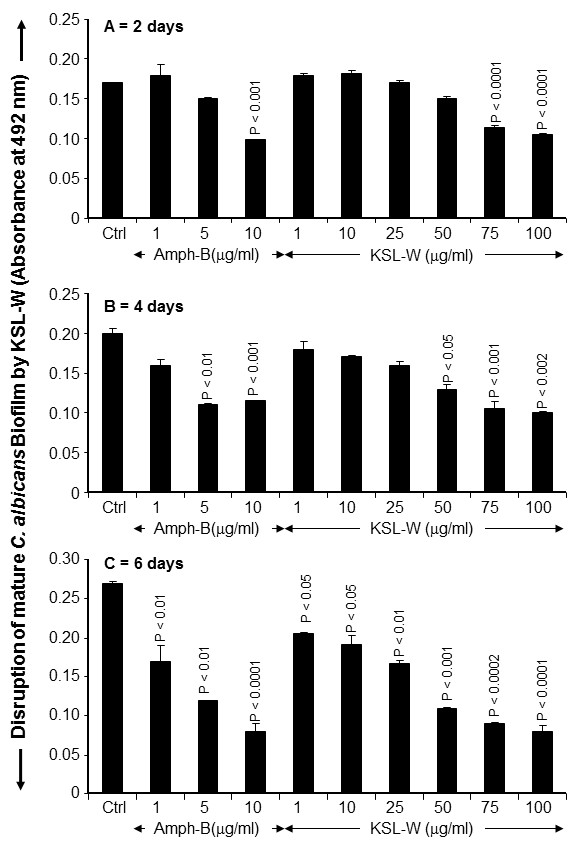
**Decrease of biofilm mass following KSL-W treatment.***C. albicans* was cultured in a 3D porous scaffold in Sabouraud medium for 6 days to promote biofilm formation and maturation. The resulting biofilms were exposed or not to KSL-W or amphotericin B for 2, 4, and 6 days. Medium and peptide were refreshed every 2 days. Following each treatment period, the samples were supplemented with XTT solution and subsequently incubated for 5 h at 37°C. Absorbance at 450 nm was measured to quantify XTT metabolic product intensity proportional to the number of viable cells. **(A)** 2 days; **(B)** 4 days; **(C)** 6 days. Results are means ± SD for three separate experiments.

After 6 days of incubation in glucose-rich Sabouraud medium, scaffolds seeded with *C. albicans* strain SC5314 produced mature biofilms displaying highly dense populations of *Candida* cells (Figure 5). Significant reductions and disruptions of the pre-formed *Candida* biofilms were observed when the reference antifungal agent (amphotericin B, 10 μg/ml) was added to the mature biofilms upon further incubation up to 6 days. Similarly, antimicrobial peptide KSL-W at 75 and 100 μg/ml also reduced *C. albicans* density in the biofilms. The observed reduction was noticed with KSL-W concentrations ranging from 25 to 100 μg/ml. Indeed, when quantitatively investigated by XTT reduction assay, the KSL-W-treated biofilms rendered a significantly lower number of cells, as reflected by the lower absorbance readings, than did the untreated control. This effect was observed after 2, 4, and 6 days of treatment with amphotericin B. Furthermore, the effect of KSL-W on the mature *C. albicans* biofilm was comparable to that obtained with amphotericin B (Figure 6).

### KSL-W modulated the expression of various *C. albicans* genes

Based on the data showing that KSL-W reduced *C. albicans* proliferation, transition, and biofilm formation, we sought to determine the involvement, if any, of gene regulation. For this purpose, we first investigated the effect of KSL-W on the activation/repression of various *C. albicans* genes when cultured under normal non-hyphae-inducing conditions. The data in Table 2 indicate that the HWP1 gene was significantly downregulated following exposure of the *C. albicans* to KSL-W for 6 h. This downregulation was comparable to that observed in the amphotericin B treatment. Similarly, SAPs 2, 4, 5, and 6 were significantly downregulated by KSL-W treatment after 6 h (Table 2). This effect was observed with both low and high concentrations of KSL-W. Furthermore, the EAP1 gene, which encodes a glycosylphosphatidylinositol-anchored, glucan-crosslinked cell wall protein in both adhesion and biofilm formation *in vitro* and *in vivo*, was also affected by the KSL-W treatment. Moreover, the expression of this gene was downregulated by KSL-W, yet was upregulated (up to 5-fold) by amphotericin B.

**Table 2:** Gene expression (6 h) under non-hyphae inducing culture conditions

**Gene**	**Untreated *C. albicans***	**Amphotericin B**		**KSL-W 25 μg/ml**		**KSL-W 100 μg/ml**	
	**Fold change**^**1**^	**Fold change**^**1**^	**p-value**^**2**^	**Fold change**^**1**^	**p-value**^**2**^	**Fold change**^**1**^	**p-value**^**2**^
*SAP2*	0.99	0.57	0.001	0.24	<0.001	0.11	<0.001
*SAP4*	0.96	0.19	<0.001	0.29	<0.001	0.14	<0.001
*SAP5*	1.00	0.08	<0.001	0.16	<0.001	0.06	<0.001
*SAP6*	1.00	0.05	<0.001	0.14	<0.001	0.04	<0.001
*EAP1*	1.00	4.91	0.028	0.4	<0.001	0.29	<0.001
*HWP1*	1.00	0.01	<0.001	0.6	0.032	0.02	<0.001

^1^Fold change was calculated by PCR product of the gene of interest/the PCR product of ACT1 (the house keeping gene), and normalized to the negative control of untreated *C. albicans* where the expression was considered equal to 1.

^2^P-values were obtained after comparison of test to negative control (untreated *C. albicans*).

Two other genes involved in regulating *C. albicans* morphogenesis, namely, *EFG1* and *NRG1*, are known to be hyphae repressors. In our study, amphotericin B increased both EFG1 and NRG1 mRNA expression, with twice as much expression for *NRG1* than for *EFG1* (Table 3), while KSL-W induced a less significant increase of *EFG1* and *NRG1* mRNA expression. Of interest is that a low KSL-W concentration (25 μg/ml) induced greater gene expression (Table 3).

**Table 3:** Gene expression (3 h) under non-hyphae inducing culture conditions

**Gene**	**Untreated *C. albicans***	**Amphotericin B**		**KSL-W 25 μg/ml**		**KSL-W 100 μg/ml**	
	**Fold change**^**1**^	**Fold change**^**1**^	**p-value**^**2**^	**Fold change**^**1**^	**p-value**^**2**^	**Fold change**^**1**^	**p-value**^**2**^
*EFG1*	1.00	5.71	<0.001	2.76	<0.001	1.98	0.073
*NRG1*	1.00	10.99	<0.001	1.77	<0.001	1.4	0.086

^1^Fold change was calculated by PCR product of the gene of interest/the PCR product of ACT1 (the house keeping gene), and normalized to the negative control of untreated *C. albicans* where the expression was considered equal to 1.

^2^P-values were obtained after comparison of test to negative control (untreated *C. albicans*).

In a second set of experiments, *C. albicans* was cultured under hyphae-inducing conditions (fetal calf serum-enriched medium with incubation at 37°C) in the presence or not of KSL-W, after which time gene expression/repression was investigated. The data in Table 4 reveal that similar to the results obtained with amphotericin-B, the *HWP1* gene was significantly (p < 0.0001) downregulated when *C. albicans* was exposed to KSL-W for 3 h, confirming the results obtained under non-hyphae growth conditions.

**Table 4:** Gene expression (3 h) under hyphae inducing culture conditions (medium supplemented with 10% fetal calf serum, with culture incubation at 37ºC)

**Gene**	**Untreated *C. albicans***	**Amphotericin B**		**KSL-W 25 μg/ml**		**KSL-W 100 μg/ml**	
	**Fold change**^**1**^	**Fold change**^**1**^	**p-value**^**2**^	**Fold change**^**1**^	**p-value**^**2**^	**Fold change**^**1**^	**p-value**^**2**^
*SAP2*	0.99	3.36	0.003	0.78	0.02	0.62	0.003
*SAP4*	0.96	2.41	0.02	0.44	0.0002	0.24	< 0.0001
*SAP5*	1.00	0.49	0.0007	0.83	0.03	0.01	< 0.0001
*SAP6*	1.00	2.56	0.01	0.30	< 0.0001	0.11	< 0.0001
*EAP1*	1.00	6.06	< 0.001	1.06	0.4	0.99	0.8
*EFG1*	1.00	1.09	0.6	0.55	0.0004	0.66	0.02
*NRG1*	1.00	2.45	0.01	0.66	0.0006	0.64	0.0005
*HWP1*	1.00	0.0055	< 0.001	0.078	< 0.0001	0.0035	< 0.0001

^1^Fold change was calculated by PCR product of the gene of interest/the PCR product of ACT1 (the house keeping gene), and normalized to the negative control of untreated *C. albicans* where the expression was considered equal to 1.

^2^P-values were obtained after comparison of test to negative control (untreated *C. albicans*).

*SAP* genes were also modulated by KSL-W treatment. Table 4 shows that after 3 h of exposure, SAPs 2, 4, 5, and 6 were significantly (p < 0.05) downregulated by the KSL-W treatment. In contrast, with amphotericin-B, a significant (p < 0.05) increase of *SAP*s 2, 4, and 6 and a decrease of *SAP*5 was observed. It is interesting to note the opposite modulatory effects of KSL-W and amphotericin-B on *SAP* gene expression. After 6 h of treatment with KSL-W, a significant decrease of each tested *SAP* gene was observed in the exposed *C. albicans*, whereas after treatment with amphotericin-B, these same *SAP* genes increased, thus confirming the antagonistic behavior of KSL-W and amphotericin-B on *SAP* gene expression.

*C. albicans EAP1* gene expression was unchanged after 3 h with KSL-W, but significantly (p < 0.001) decreased after 6 h, while the expression of this gene was upregulated (close to six folds) by amphotericin B (Tables 4 and 5). Amphotericin B increased NRG1 mRNA expression almost threefold, with no significant effect on the *EFG1* gene, yet significantly decreased HWP1 gene expression. On the other hand, after 3 h (Table 4) and 6 h (Table 5) of incubation, KSL-W downregulated EFG1, NRG1, and HWP1 mRNA expression. Of interest is that except for similar downregulatory effects on *HWP1* gene expression, KSL-W and amphotericin-B produced once again opposite results regarding *EFG1*and *NRG1* gene expression.

**Table 5:** Gene expression (6 h) under hyphae inducing culture conditions (medium supplemented with 10% fetal calf serum, with culture incubation at 37ºC)

**Gene**	**Untreated *C. albicans***	**Amphotericin B**		**KSL-W 25 μg/ml**		**KSL-W 100 μg/ml**	
	**Fold change**^**1**^	**Fold change**^**1**^	**p-value**^**2**^	**Fold change**^**1**^	**p-value**^**2**^	**Fold change**^**1**^	**p-value**^**2**^
*SAP2*	0.99	8.17	0.009	0.7	0.2	1.31	0.02
*SAP4*	0.96	2.58	0.03	0.73	0.04	0.72	0.04
*SAP5*	1.00	0.72	0.007	0.83	0.0004	0.56	0.006
*SAP6*	1.00	4.01	0.02	0.58	0.01	0.68	0.04
*EAP1*	1.00	6.36	0.001	0.44	0.008	0.73	0.003
*EFG1*	1.00	1.78	0.048	0.31	< 0.0001	0.47	0.01
*NRG1*	1.00	3.97	0.0005	0.37	0.001	0.37	0.05
*HWP1*	1.00	0.008	< 0.001	0.09	0.001	0.03	< 0.0001

^1^Fold change was calculated by PCR product of the gene of interest/the PCR product of ACT1 (the house keeping gene), and normalized to the negative control of untreated *C. albicans* where the expression was considered equal to 1.

^2^P-values were obtained after comparison of test to negative control (untreated *C. albicans*).

## Discussion and conclusions

We demonstrated that KSL-W was effective in inhibiting *C. albicans* growth at short and long culture periods. Although growth inhibition obtained with KSL-W was less than that obtained with amphotericin B, the effects of KSL-W nevertheless remain significant (p < 0.01). The growth inhibition effects of KSL-W are in accordance with previously reported findings [37] showing a downregulation of *C. albicans* activity induced by a bacteriocin-like peptide isolated from *Lactobacillus pentosus*. Furthermore, our results support other findings [38] reporting the effectiveness of KSL-W in disrupting *P. gingivalis-*induced hemagglutination and its synergistic interaction with host AMPs engaged in innate defense. The results strongly suggest that KSL-W is also effective against fungal growth and may be suitable for use to control *C. albicans* infections. Further studies on the possible synergistic effect of amphotericin B and KSL-W against *C. albicans* growth may provide insight.

*C. albicans* pathogenesis can also take place through the transition from blastospore to hyphal form [39,40]. Our results indeed show that KSL-W completely inhibited *C. albicans* transition with a concentration as low as 5 μg/ml. These data are consistent with those of other studies with naturally occurring antimicrobial peptides (e.g., β-defensins) which were effective in blocking the morphological shift of *Candida* from yeast to hyphae [41,42]. Thus KSL-W may possibly contribute to the control of *C. albicans* infection by reducing cell growth and yeast-hyphae transition. The effect of KSL-W on *C. albicans* growth can occur either through cytolysis or cell membrane disruption, resulting in cell death similar to what has been demonstrated with histatin-5 [43,44]. Indeed, it was shown that histatin-5 induces the selective leakage of intracellular ions and ATP from yeast cells. This is caused by the translocation of histatin-5 into the intracellular compartment and accumulates to a critical concentration [45]. Further studies are thus warranted to shed light on the fungicidal mechanism of KSL-W.

*C. albicans* growth and transition from blastospore to hyphal form are particularly important for biofilm formation and *C. albicans* virulence because a strain that is genetically manipulated to grow exclusively in the yeast form is greatly hindered in generating biofilms. In addition, a variety of *C. albicans* mutants known to be unable to form hyphae also show biofilm defects [46,47]. As KSL-W significantly reduced *C. albicans* growth and inhibited its transition from yeast to hyphae, this suggests that KSL-W may inhibit *C. albicans* biofilm formation. Our findings indicate that KSL-W was indeed able to reduce biofilm formation and that its effect was comparable to that obtained with amphotericin B, a well-known antifungal molecule. Also of interest is that a significant inhibition of *C. albicans* biofilm formation was obtained at a concentration of as low as 25 μg/ml of KSL-W antimicrobial peptide. These useful data are comparable to those of other studies showing the positive action of synthetic peptide in controlling and preventing microbial biofilm formation [48]. Thus, with its significant impact in reducing *C. albicans* biofilm formation, KSL-W may show potential for several novel applications in the clinical setting. Further investigations will elucidate this effect.

Biofilm formation can be controlled with anti-biofilm molecules prior to its development, although this is not actually the case in clinical applications, as antifungal and microbial molecules cannot be used on a daily basis to prevent biofilm formation. An effective molecule should ideally be able to prevent biofilm formation, but more importantly to disrupt biofilms that are already formed. We therefore questioned whether KSL-W was capable of disrupting mature *C. albicans* biofilm.

We proceeded to examine the impact of KSL-W on mature biofilm formation and demonstrated a significant disruption of these biofilms following contact with KSL-W, thus suggesting the possible use of this antimicrobial peptide to reduce/eliminate mature biofilms. Further studies should confirm such observations and demonstrate how KSL-W reduces or disrupts *C. albicans* biofilms.

Once it reaches the cell, KSL-W can potentially act on the cytoplasmic membrane as well as on intracellular targets [49-51]. The action of KSL-W against *C. albicans* may operate through the modulated expression of certain *C. albicans* genes that control growth [52], transition [53], and biofilm formation [54]. We therefore examined the effect of KSL-W on a number of genes either directly or indirectly involved in phase transition and biofilm formation. *EFG1* and *NRG1* expression was assessed under hyphae/non-hyphae-inducing conditions. Our results show that KSL-W increased NRG1 mRNA expression twofold under non-hyphae-inducing conditions; however, under hyphae-inducing conditions, KSL-W significantly reduced *NRG1* gene expression. These findings contrast with other reports that an increased NRG1 expression contributes to repressing various hypha-specific genes [55,56]. This confirms that the effect of KSL-W in controlling *C. albicans* virulence does not take place through NRG1. KSL-W was also able to decrease EFG1 mRNA expression, when *C. albicans* was maintained under hyphae-inducing conditions.

EFG1p has been found to be a central regulator of *C. albicans*, as it is required for the development of a true hyphal growth form, and EFG1 is considered to be essential in the interactions between *C. albicans* and human host cells [7,8]. The downregulation of this gene by KSL-W points to the singular role of this antifungal peptide. Thus the effect of KSL-W on *C. albicans* transition can be manifested through a repression of certain genes, such as *EFG1* and *NRG1*.

KSL-W has a significant inhibitory effect on EAP1 mRNA expression. As a member of the GPI-CWP family [5,57], deleting EAP1 can reduce the adhesion of *C. albicans* to different surfaces. This suggests that treatment with KSL-W may reduce EAP1 expression, which in turn may contribute to reducing *C. albicans* adhesion and ultimately, biofilm formation and pathogenesis. KSL-W was also shown to reduce HWP1 mRNA expression, particularly when *C. albicans* was cultured under hyphae-inducing conditions.

HWP1 is a downstream component of the cAMP-dependent PKA pathway and is positively regulated by EFG1 [58]. The transcript level of HWP1 decreased with the KSL-W treatment at low and high concentrations. These data suggest that KSL-W indeed impacts the activity of the cAMP–EFG1 pathway and leads to an alteration of *C. albicans* growth and morphogenesis. Further studies are therefore required to investigate the invasion/virulence of KSL-W-treated *C. albicans*.

It is well known that *Candida* pathogenesis can be established by virtue of *Candida* growth and yeast-to-hyphae morphogenesis. Specific *SAP* genes were found to be preferentially expressed by *Candida* hyphal forms [10,15,59]. Because KSL-W downregulated *C. albicans* growth and transition, this may have occurred through a modulation of the *SAP* genes. Our findings confirm that KSL-W is capable of decreasing SAP2, SAP4, SAP5, and SAP6 mRNA expression in *C. albicans* which may lead to reducing *C. albicans* virulence [60-62].

Our study thus establishes, for the first time, a clear link between an antimicrobial peptide (KSL-W), hyphae morphogenesis, and hyphae-modulating SAPs 2, 4, 5, and 6. However, the precise interactions between these SAPs and KSL-W during *C. albicans* pathogenesis remain unclear. Additional studies should focus on identifying the role of SAP subfamilies involved in *Candida* invasion as well as the role of KSL-W in controlling *Candida* virulence/pathogenesis in conjunction with host defenses. In conclusion, this study is the first to demonstrate that synthetic antimicrobial peptide KSL-W downregulates *C. albicans* growth and transition, resulting in a decrease in biofilm formation and a disruption of mature biofilm. Also of interest is that these effects may occur through the modulation of *C. albicans* genes *EFG1*, *NRG1*, *EAP1*, *HWP1*, and *SAP*s. Overall results clearly suggest the potential of KSL-W as an antifungal molecule.

## Methods

### *C. albicans*

*C. albicans* strain ATCC-SC5314 was cultured for 24 h on Sabouraud dextrose agar plates (Becton Dickinson, Oakville, ON, Canada) at 30°C. For the *C. albicans* suspensions, one colony was used to inoculate 10 ml of Sabouraud liquid medium supplemented with 0.1% glucose at pH 5.6. The cultures were grown overnight in a shaking water bath for 18 h at 30°C. The yeast cells were then collected, washed with phosphate-buffered saline (PBS), counted with a haemocytometer, and adjusted to 10^7^/ml prior to use.

### Antimicrobial peptides

KSL-W (KKVVFWVKFK-NH2) was synthesized by standard solid-phase procedures [63] with 9-fluorenylmethoxycarbonyl (Fmoc) chemistry in an automatic peptide synthesizer (model 90, Advanced ChemTech, Louisville, KY, USA). The synthetic peptides were then purified by reverse-phase HPLC (series 1100, Hewlett Packard) by means of a Vydac C18 column. Peptide purity was confirmed by MALDI-TOF (matrix-assisted laser desorption/ionization-time of flight) MS (AnaSpec Fremont, CA, USA). The final product was stored in lyophilized format -20°C until use. KSL-W solution was prepared, filtered (0.22 um pore size), and used for the experiments. Amphotericin B (Sigma-Aldrich, St. Louis, MO, USA) was dissolved in distilled water to obtain a 250 μg/ml concentration which was also filtered, with the sterile solution stored at -80°C until use.

### Effect of KSL-W on *C. albicans* proliferation

Proliferation was investigated by placing 10^4^*C. albicans* in 200 μL of Sabouraud dextrose broth in a round-bottom 96-well plate. The *C. albicans* cultures were supplemented with KSL-W at concentrations of 1, 10, 25, 50, 75, and 100 μg/ml. The negative controls were *C. albicans* cultures not supplemented with KSL-W, while the positive controls were *C. albicans* cultures supplemented with amphotericin B at concentrations of 1, 5, and 10 μg/ml. The plates were incubated for 5, 10, and 15 h prior to cell growth analyses. *C. albicans* growth was assessed using the (3-(4,5-dimethylthiazol-2-yl)-2,5-diphenyl tetrazolium bromide) MTT assay (Sigma-Aldrich) which measures cell growth as a function of mitochondrial activity [64]. Briefly, an MTT stock solution (5 mg/ml) was prepared in PBS and added to each culture at a final concentration of 10% (v/v). The *C. albicans* cultures were then incubated with the MTT solution at 30°C for 4 h, after which time the plate was centrifuged for 10 min at 1200 rpm and the supernatant was removed. The remaining pellet from each well was then washed with warm PBS, with 200 μl of 0.04 N HCl in isopropanol added to each well, followed by another incubation for 15 min. Absorbance (optical density, OD) was subsequently measured at 550 nm by means of an xMark microplate spectrophotometer (Bio-Rad, Mississauga, ON, Canada). Results are reported as means ± SD of three separate experiments.

### Effect of KSL-W on *C. albicans* transition from blastospore to hyphal form

To determine the effect of KSL-W on the yeast-to-hyphae transition, *C. albicans* (10^5^ cells) was first grown in 500 ml of Sabouraud dextrose broth supplemented with 0.1% glucose and 10% fetal bovine serum (FBS). KSL-W was then added (or not) to the culture at various concentrations (1, 5, 10, 15, and 25 μg/ml). The negative controls were the *C. albicans* cultures without antimicrobial peptide, while the positive controls represented the *C. albicans* cultures supplemented with amphotericin B (1, 5, and 10 μg/ml). The hyphae-inducing conditions were previously reported [65], consisting of culture medium supplementation with 10% fetal calf serum and subsequent incubation at 37°C. These conditions were used in our experiments. Following incubation for 4 or 8 h, the cultures were observed microscopically and photographed to record *C. albicans* morphology (n = 5) and the density of *C. albicans* transition was measured.

### Effect of KSL-W on *C. albicans* gene activation/repression

*C. albicans* was subcultured overnight in Sabouraud liquid medium supplemented with 0.1% glucose, pH 5.6, in a shaking water bath for 18 h at 30°C. The yeast cells were then collected, washed with PBS, and counted with a hemocytometer, after which time they were co-cultured with or without the antimicrobial peptide under hyphae- or non-hyphae-inducing conditions, as follows.

#### Effect of KSL-W on gene activation when C. albicans was cultured under non-hyphae-inducing conditions

*C. albicans* was co-cultured with either KSL-W (1, 25, 100 μg/ml) or amphotericin B (1 μg/ml) or with none of these molecules (controls) in Sabouraud liquid medium supplemented with 0.1% glucose, pH 5.6. The cultures were maintained at 30°C for 3 and 6 h.

#### Effect of KSL-W on gene activation when C. albicans were cultured under hyphae-inducing conditions

*C. albicans* was co-cultured with either KSL-W (1, 25, 100 μg/ml) or amphotericin B (1 μg/ml) or with none of these molecules (controls) in Sabouraud liquid medium supplemented with 0.1% glucose, pH 5.6. As previously reported, to promote the transition of *C. albicans* from blastospore to hyphal form, the culture medium was supplemented with 10% fetal calf serum and the incubation was performed for 3 and 6 h at 37°C. Following each culture period under both conditions [68], the cultures were centrifuged 10 min at 13,000 rpm, the supernatants were discarded, and each pellet was suspended thereafter in 0.6 ml of lysis buffer (Glycerol 1 M, EDTA 0.1 M). Glass beads (0.425-0.6 mm in diameter; 0.2 ml) were added to each suspended pellet prior to sonication (4 × 1 min, followed by 2 min of incubation in ice) with a MiniBead-beater (Biospec Products, Bartlesville, OK, USA). Following cell lysis, the total RNA was extracted from each sample by means of the Illustra RNAspin Mini kit (GE Health Care UK Limited, Buckingham, UK). Concentration, purity, and quality of the isolated RNA were determined using the Experion system and RNA StdSens analysis kit according to the instructions provided by the manufacturer (Bio-Rad, Hercules, CA, USA).

### Quantitative real-time RT-PCR

The RNA (500 ng of each sample) was reverse transcribed into cDNA by means of the iScript cDNA Synthesis kit (Bio-Rad, Mississauga, ON, Canada). The conditions for the preparation of the cDNA templates for PCR analysis were 5 min at 25°C, 1 h at 42°C, and 5 min at 85°C. Quantitative PCR (qPCR) was carried out as previously described [36]. The quantity of mRNA transcripts was measured with the Bio-Rad CFX96 real-time PCR detection system. Reactions were performed using a PCR supermix, also from Bio-Rad (iQ SYBR Green supermix). Primers (Table 6) were added to the reaction mix to a final concentration of 250 nM. Five microliters of each cDNA sample were added to a 20 μl PCR mixture containing 12.5 μl of the iQ SYBR Green supermix, 0.5 μl of specific primers ACT1*,* SAP2, SAP4, SAP5, SAP6, HWP1, and EAP1 (Midland Certified Reagent Company, Inc., Midland, TX, USA), as well as EFG1 and NRG1 (Invitrogen Life Technologies Inc., Burlington, ON, Canada), and 7 μl of RNase/DNase-free water (MP Biomedicals, Solon, OH, USA). Each reaction was performed in a Bio-Rad MyCycler Thermal Cycler. For the qPCR, the CT was automatically determined using the accompanying Bio-Rad CFX Manager. The thermocycling conditions for the ACT1, SAPs 2-4-5-6, and EAP1 were established as 5 min at 95°C, followed by 30 cycles of 15 s at 95°C, 30 s at 60°C, and 30 s at 72°C, with each reaction performed in triplicate. For the EFG1 and NRG1, the thermocycling conditions were set for 3 min at 95°C, followed by 45 cycles of 10 s at 95°C, 40 s at 54°C, and 40 s at 72°C, with each reaction also performed in triplicate. For the HWP1, the conditions were 3 min at 95°C, followed by 45 cycles of 10 s at 95°C, 30 s at 54°C, and 40 s at 72°C, with each reaction performed in triplicate. The specificity of each primer pair was determined by the presence of a single melting temperature peak. The ACT1 produced uniform expression levels varying by less than 0.5 CTs between sample conditions and thus became the reference gene for this study. The results were analyzed by means of the 2^−ΔΔCt^ (Livak) relative expression method.

**Table 6:** Primer sequences used for the qRT-PCR

**Gene**	**Primer sequence 5′ to 3′**	**Amp size (bp)**
***ACT1***	Forward : GCTGGTAGAGACTTGACCAACCA	87
	Reverse : GACAATTTCTCTTTCAGCACTAGTAGTGA	
***SAP2***	Forward : TCCTGATGTTAATGTTGATTGTCAAG	82
	Reverse : TGGATCATATGTCCCCTTTTGTT	
***SAP4***	Forward : AGATATTGAGCCCACAGAAATTCC	82
	Reverse : CAATTTAACTGCAACAGGTCCTCTT	
***SAP5***	Forward : CAGAATTTCCCGTCGATGAGA	78
	Reverse : CATTGTGCAAAGTAACTGCAACAG	
***SAP6***	Forward : TTACGCAAAAGGTAACTTGTATCAAGA	102
	Reverse : CCTTTATGAGCACTAGTAGACCAAACG	
***ALS3***	Forward : AATGGTCCTTATGAATCACCATCTACTA	51
	Reverse : GAGTTTTCATCCATACTTGATTTCACAT	
***HWP1***	Forward : GCTCAACTTATTGCTATCGCTTATTACA	67
	Reverse : GACCGTCTACCTGTGGGACAGT	
***EAP1***	Forward : CTGCTCACTCAACTTCAATTGTCG	51
	Reverse : GAACACATCCACCTTCGGGA	
***EFG1***	Forward : TATGCCCCAGCAAACAACTG	202
	Reverse : TTGTTGTCCTGCTGTCTGTC	
***NRG1***	Forward : CACCTCACTTGCAACCCC	198
	Reverse : GCCCTGGAGATGGTCTGA	

### Effect of KSL-W on *C. albicans* biofilm formation

*C. albicans* biofilms were obtained by culturing the yeast on a porous collagen scaffold which facilitated *C. albicans* penetration through the pores and its adhesion to the scaffold through collagen affinity. This also promoted biofilm formation and handling with no cell loss, thus contributing to maintaining the biofilm structure. For this purpose, 5 mm × 5 mm samples of porous scaffold (Collatape, Zimmer Dental Inc., Carlsbad, CA, USA) were placed into a 24-well plate. The scaffolds were then rinsed twice with culture medium, seeded with *C. albicans* (10^5^ cells), and incubated for 30 min at 30°C without shaking to allow for adherence. Fresh Sabouraud medium was added to each well in the presence or absence of various concentrations of KSL-W (1, 10, 25, 50, 75, and 100 μg/ml). Two controls were included in this study: the negative control was *C. albicans* seeded without KSL-W, while the positive control was *C. albicans* seeded with amphotericin B (1, 5, and 10 μg/ml). The *C. albicans*-seeded scaffolds were then incubated for 2, 4, and 6 days at 30°C. The medium, KSL-W, and amphotericin B were refreshed every 48 h. Following each culture period, *C. albicans* growth and biofilm formation was assessed by scanning electron microscopy and XTT-menadione assay.

#### Scanning electron microscopy (SEM) analysis

Biofilms were fixed in ethylene glycol for 60 min and rinsed once with sterile PBS. Dehydration was performed in a series of 5-min treatments with ethanol solutions of increasing concentration (50, 70, 90, and twice at 100%). The dehydrated biofilms were kept overnight in a vacuum oven at 25°C, after which time they were sputter-coated with gold, examined, and imaged (n = 4) under a JEOL 6360 LV SEM (Soquelec, Montréal, QC, Canada) operating at a 30 kV accelerating voltage.

#### XTT reduction assay

To support the hypothesis that KSL-W quantitatively affects *C. albicans* biofilms, an XTT reduction assay was performed on the KSL-W-treated and control biofilms at defined time points. XTT assay is one of the most useful and accurate methods to investigate microbial biofilm formation. The metabolic activity of the biofilm cells was measured as a reflection of viable cell count. To do so, *C. albicans* biofilms formed in the porous scaffold with or without KSL-W treatments for 2, 4, and 6 days were subjected to an XTT assay. Fifty microliters of XTT salt solution (1 mg/ml in PBS; Sigma-Aldrich) and 4 μl of menadione solution (1 mM in acetone; Sigma-Aldrich) were added to wells containing 4 ml of sterile PBS. The biofilms were then added to the mixture and the plates were incubated at 37°C for 5 h, after which time the supernatant was collected to measure the XTT formazan at 492 nm by means of an xMark microplate spectrophotometer (Bio-Rad, Mississauga, ON, Canada).

### Effect of KSL-W on the disruption of mature *C. albicans* biofilms

Mature *C. albicans* biofilms were obtained by culturing *C. albicans* (10^5^) on a porous 3D collagen scaffold for 6 days at 30°C in Sabouraud liquid medium supplemented with 0.1% glucose at pH 5.6. The culture medium was refreshed every 2 days. At the end of the 6-day culture period, the biofilms were treated (or not) with KSL-W (75 and 100 μg/ml). Amphotericin B-treated biofilms (1, 5, and 10 μg/ml) were used as the positive controls. The biofilms were continuously incubated (or not) with either KSL-W or amphotericin B for 2, 4, and 6 days, with medium changing every day. KSL-W and amphotericin B were also refreshed at each medium changing. Following each incubation period, SEM and XTT analyses were performed, as described above.

### Statistical analysis

Each experiment was performed at least four times, with experimental values expressed as means ± SD. The statistical significance of the differences between the control (absence of KSL-W) and test (presence of KSL-W or amphotericin B) values was determined by means of a one-way ANOVA. Posteriori comparisons were performed using Tukey’s method. Normality and variance assumptions were verified using the Shapiro-Wilk test and the Brown and Forsythe test, respectively. All of the assumptions were fulfilled. P values were declared significant at ≤ 0.05. The data were analyzed using the SAS version 8.2 statistical package (SAS Institute Inc., Cary, NC, USA).

## Authors’ contributions

MR, KPL, and AS designed the experiments, supervised the research and wrote the paper. ST, AA, and AS performed the experiments and data analyses and contributed to the writing of the paper. Each author read and approved the final manuscript.
